# Early Onset Scoliosis and Adolescent Idiopathic Scoliosis: A Review of the Literature and Correlations With Pulmonary Dysfunction

**DOI:** 10.7759/cureus.48900

**Published:** 2023-11-16

**Authors:** Emmanuel K Mbamalu, Julia Hyacinthe, Aaron Hui, Parsa Tirabady, Leila Alvandi, Jaime Gomez

**Affiliations:** 1 Orthopaedic Surgery, Albert Einstein College of Medicine, New York, USA; 2 Orthopaedic Surgery, Montefiore Medical Center, New York, USA

**Keywords:** quality of life, obstructive lung disease, restrictive lung disease, thoracic cage, pulmonary function tests, adolescent idiopathic scoliosis, early onset scoliosis

## Abstract

In the management of early onset scoliosis (EOS) and adolescent idiopathic scoliosis (AIS), orthopedic surgeons are tasked with considering the effects that curves and their treatment can have on the respiratory system, possibly the most relevant being pulmonary dysfunction due to thoracic cage changes. The pulmonary impairment that occurs as a result of scoliosis varies widely and requires a multimodal response, including physiologic testing, such as pulmonary function tests (PFTs) and consistent psychosocial monitoring of the patient. This forces healthcare providers to consider all factors affecting the patient's quality of life (QOL) and not just the primary pathology they are treating. One method that could be utilized to ensure a more holistic approach to treatment is the use of patient-reported outcome measures (PROMs) to assess the QOL domains. Thus, this review serves to highlight the importance of addressing and correcting pulmonary dysfunction in the care of children with EOS and AIS in a holistic manner.

## Introduction and background

Scoliosis, defined as an abnormal lateral curvature of the spine of at least 10 degrees in the coronal plane, can be categorized by etiology: idiopathic, congenital, or neuromuscular [1]. Idiopathic scoliosis (IS) is present in about 2-3% of children and represents 80-85% of all pediatric spinal deformity cases [2,3]. It can be further categorized into infantile, juvenile, and adolescent based on the age of onset. Infantile scoliosis has an age of onset of three years or younger, juvenile presents between the ages of four and nine, and adolescent occurs between the ages of 10 and 18 [4]. Adolescent idiopathic scoliosis (AIS) is the most common form of pediatric scoliosis; however, its causes remain unknown [1]. Genetic factors are thought to be involved, as nearly 30% of patients with AIS have family members with scoliosis. In addition, IS disproportionately affects females more than males and prefers right-sided curvature. Curves measuring over 40 degrees are only present in approximately 0.1% of patients with AIS, but these tend to be the cases that require surgical interventions [1]. Congenital scoliosis occurs as a result of congenital disturbances in vertebral formation and segmentation, while neuromuscular scoliosis occurs secondary to neuromuscular conditions, such as muscular dystrophy or cerebral palsy [5]. Early onset scoliosis (EOS) is defined as scoliosis of any etiology that occurs before the age of 10 [1,6]. The prevalence of EOS is lower than that of AIS, as it accounts for only about 10% of all pediatric scoliosis cases, and it occurs more commonly in males than females [5,7]. EOS also typically has a more complex and challenging clinical picture than AIS given that younger patients' spines and thoraxes are impacted at critical stages of growth and development [5]. 

Historically, the greatest concern in the treatment of children with EOS and AIS has been preserving and maximizing the growth potential of the spine while delaying its curve progression [8]. The goal is delaying surgery, if not eliminating the need for surgery all together [9,10]. In attempting to correct and guide the growth of the spine in these children, caregivers are working to ensure the development of a thoracic cavity with enough volume to house all necessary anatomical structures with adequate space [6]. The volume of the thoracic cavity is directly affected by the orientation of the vertebral column and spinal deformities are known to inhibit normal development of the thorax by causing a constriction in its three-dimensional area [11]. One of the most pressing concerns in the care of children with EOS and AIS is that the constriction in thoracic cavity volume has the potential to significantly impair pulmonary function, with increased risk for morbidity and early mortality [12,13]. As a part of the process of overcoming this obstacle facing EOS and AIS patients, this review aims to compile and summarize the current research on lung dysfunction in children with EOS and AIS and encourage further research and discourse in the care of this patient population.

## Review

Pathogenesis of IS and pathophysiology of lung dysfunction 

The role of genetic influence in IS has been well documented, but the specific mechanisms have yet to be determined [14]. Although the inheritance pattern has not yet been defined, multiple studies have shown that the risk of IS quickly declines when comparing first-degree relatives of a proband to later generations [14]. One particular population study found that the risk to first-degree relatives was 11%, while in second- and third-degree relatives, the risk was 2.4% and 1.4%, respectively [15]. Furthermore, severity and susceptibility of IS have been correlated in monozygotic twins, but the curve pattern has not, and concordances in monozygotic twins were found to be below 100% [14]. The findings to date may be evidence of a multifactorial inheritance pattern that is further influenced by certain environmental factors in what can be considered a complex model of genetic disease. In addition, studies attempting to identify specific genes associated with IS have been unrevealing and generally nonspecific as of yet [14]. 

In addition to genetic factors, certain proteins and hormones have been found to play a potential role in the pathogenesis of AIS [16]. Some studies have shown that girls with AIS have lower serum levels of estradiol, implying that sex hormones may play a role in the disease. Other studies have shown a relationship between scoliosis progression and increased levels of platelet calmodulin, which may be due to calmodulin acting as a mediator of contractile tissues, impacting paraspinous muscle activity [16]. Melatonin has also been implicated in the disease process of scoliosis, with levels of the hormone being significantly lower in AIS patients. This may also play a role in the effects of calmodulin, as there are interactions between calmodulin and melatonin [16]. Continued efforts must be invested by the scientific community to understand the genetic basis and general pathogenesis of the disease, as doing so can have profound implications on diagnostic and treatment possibilities.

Scoliosis typically presents with trunk and waistline asymmetry, unequal shoulder levels, and rib prominence, with rightward thoracic curves being more prominent in AIS [16]. Patients can also have associated back pain, although it is a relatively uncommon finding. Young people with AIS are also typically quite active and tend to be otherwise healthy children [1]. A retrospective analysis by McElroy et al. found that significant differences exist between operative EOS and AIS curves, with those in EOS being greater in magnitude, more kyphotic, and less well compensated [17]. The more severe clinical picture seen in EOS is, as aforementioned, likely due at least in part to the younger age of onset. Patients with a Cobb angle greater than 70 are typically those that can present with pulmonary disease, and curves of this severity usually have an onset prior to 10 years of age [13,18].

Scoliosis is often perceived as an isolated pathology of the spine; however, the vertebral column also offers structural support throughout the whole trunk of the body. As such, spinal deformities, such as scoliosis, with progressive courses, thus decrease longitudinal growth and lead to an abnormal proportionality of trunk growth. This progressive impairment of normal trunk growth has been previously described as a “domino effect” [11,19]. Not only is spinal growth affected, but the size and shape of the thoracic cage are also diminished because of scoliosis, further leading to a stunting of standing and sitting height, thoracic cage shape, volume and circumference, and lung development [20]. These effects on growth, posture, and development of the trunk lead to increased risk of complications, such as thoracic insufficiency syndrome (TIS) and cor pulmonale, among other things [21,22]. TIS is defined as the inability of the chest to support normal breathing and/or lung growth and can develop secondary to the restriction and deformity caused by severe or untreated scoliosis [21]. The restrictive disease caused by scoliosis can also lead to pulmonary hypertension in severe cases, which, in turn, can affect right heart function resulting in cor pulmonale [23]. Furthermore, EOS patients often face a more complex pathophysiology than AIS patients, with EOS patients having a higher risk of developing severe scoliosis with increased morbidity and mortality compared to those with AIS [24]. This is because the earlier the patient develops EOS, the more extreme the spinal and thoracic deformity occurs. As a result, EOS patients maintain higher risk of morbidity due to further deformation than AIS patients. Because spinal growth occurs in unison with the thorax, the rapid increase in Cobb’s angle affects the thoracic cavity and thus the lungs at their most crucial stage of development. In these patients, lung hypoplasia is common, and in the progression of EOS, cardiovascular disease becomes prominent [25].

The exact mechanism by which lung dysfunction arises in children with scoliosis is mostly attributed to the restrictive pattern of lung disease [26]. Tsiligiannis and Grivas [26] described four general principles that scoliosis has on the impairment of pulmonary function: (1) a Cobb angle greater than 90 degrees greatly predisposes to cardiorespiratory failure, (2) lung function abnormalities are detectable when a Cobb angle is greater than 50 to 60 degrees, (3) lung function abnormalities are mainly of the restrictive type and (4) the duration of scoliosis correlates with the patient’s degree of disability. It should be noted that although the restrictive pattern of lung dysfunction is most prevalent in children with scoliosis, obstructive or mixed lung disease with moderate to severe air trapping has also been reported in a significant percentage of patients undergoing preoperative evaluation for scoliosis surgery [27,28]. Thus, IS can present with lung dysfunction in a restrictive, obstructive, or mixed manner depending on the individual disease progression of the patient.

In addition, children with EOS and AIS who are afflicted by restrictive lung disease experience a decrease in lung volume, which is multifactorial and may reflect individual pathophysiology [26]. EOS patients, who are at an earlier and physiologically critical stage of lung development, are more susceptible to deformations of the spine and thoracic cage [6]. Furthermore, the growth of alveoli occurs most rapidly in the first eight years of life, during which there is an exponential increase in the total number of alveoli [25]. If the thoracic deformity occurred during this period of rapid lung growth and development, true lung hypoplasia can result, which may be a factor in both infantile and juvenile scoliosis [29-31]. Through postmortem quantitative studies, it is thought that alveolar multiplication is decreased in EOS and alveoli may not enlarge normally in AIS [27]. The reduced lung volume due to restrictive disease caused by scoliosis therefore manifests as a decrease in the total lung capacity (TLC) on pulmonary function tests (PFTs) [26]. Correcting and avoiding this decrease in TLC have now become central in the treatment of children with EOS and AIS.

In a case-control study conducted by Martínez-Llorens et al., it was found that in patients with AIS, obstructive abnormalities were present in a non-negligible number of individuals (14% if both pure obstructive and mixed ventilatory patterns are taken into account) [32]. Although obstructive abnormalities are thought to be less prevalent, they may still influence chest deformities and/or respiratory muscle dysfunction on the cross-sectional area of the airways [20,32-34]. However, in the currently available literature, there remain differing opinions on the pathophysiology of obstructive and mixed lung disease in EOS and AIS [35]. This knowledge gap regarding the full etiology of lung dysfunction in scoliosis patients must be closed to develop novel methods to best correct these anatomical and physiological abnormalities. 

Assessment of pulmonary function in IS

From a quantitative standpoint, the most useful parameters in assessing lung function in children with scoliosis are PFTs. PFTs are an invaluable tool in the investigation and monitoring of scoliosis patients with respiratory pathology and can be performed as early as age four to five. They provide important information relating to the large and small airways, the pulmonary parenchyma, and the size and integrity of the pulmonary capillary bed [36]. Despite this, some recent studies have contrasting opinions on the usefulness of routinely ordering PFTs in the treatment of AIS and EOS.

A retrospective observational study conducted by Burjek et al. suggests that contrary to common practice, routine ordering of PFTs for pediatric patients with scoliosis undergoing posterior spinal fusion may not be necessary. They did not find evidence of a significant association between preoperative PFT results and postoperative outcomes. Instead, they described an association between postoperative outcomes and indicators of scoliosis severity (Cobb angle), patient medical complexity (ASA physical status, preoperative bilevel positive airway pressure support use, and cognitive dysfunction precluding PFT attempts), and magnitude of surgical insults (intraoperative allogeneic blood transfusion) [37]. Interestingly enough, results similar to those present in the Burjek et al.'s study seem to be outliers, and it is generally agreed upon that PFTs provide some degree of use in risk assessment and preoperative optimization.

Despite the limited amount of literature regarding pulmonary dysfunction in children with EOS and AIS, some current literature evaluated PFTs in the setting of surgical correction in children with EOS and AIS [36-46]. In terms of available surgical techniques, they can be divided into distraction, guided growth, or compression-based implants. Distraction-based implants are commonly used and can be attached to the spine, ribs, or pelvis. Gomez et al. described two forms of distraction-based surgical correction through growing rods or vertical expandable prosthetic titanium ribs (VEPTRs) [38]. In a retrospective observational study conducted by Chang et al., they found that there was no statistically significant increase in forced expiratory volume in one second (FEV1) and forced vital capacity (FVC) (an increase by 3% and 4% in both parameters, respectively) after repeated traditional growing rod procedures. In addition, almost 30% of patients exhibited a decline in the percentage of predicted FEV1 and FVC at the last follow-up [39]. PFTs also serve as useful practical parameters in evaluating post-treatment management of patients. For example, an FVC of less than 40% of the predicted normal and maximal inspiratory and expiratory pressures of less than 30 cm H_2_O significantly increase the risk that the patient may not be able to be extubated [26,40,41].

In a longitudinal study following children with EOS conducted by Motoyama et al., they demonstrated definite signs of lung growth following serial VEPTR thoracoplasty. However, the percent predicted values of FVC changed minimally (increased by 1.1%). Specific compliance (a measure of the lung’s ability to stretch and expand) was also abnormally decreased in some patients, indicating abnormally stiff chest walls. The average specific compliance measurements during the last tests were not significantly different from the initial measurements that were near the lower normal limit prior to VEPTR. They then conducted a follow-up study in which they found that eight of the 24 patients had an FVC 60% below predicted, indicating severe restrictive defects in the pulmonary function [43]. Results like those in this study have also been described by other groups [42,44]. These studies provide evidence for supporting that PFTs should be incorporated in modifying the treatment of children with EOS and AIS.

Treatment and outcomes for pulmonary dysfunction in IS

In the past, the main outcome of interest in treating scoliosis is correcting the curvature of the spine. In the last few decades, orthopedic surgeons have begun considering the effects of scoliosis on the thoracic cage as a whole, and the central principles of treatment for EOS and AIS changed from a primarily spine centered focus to a relatively more holistic perspective. Historically, spinal fusion was performed in children with progressive EOS, which corrected scoliosis but limited spine and thoracic growth and resulted in poor pulmonary outcomes [6]. 

Although spinal fusion is typically the first-line surgical intervention for severe scoliosis, a variety of other surgical intervention techniques exist for the management of scoliosis. Surgical techniques can be categorized as growth-friendly, definitive spinal fusion, or combinations [47]. Within the growth-friendly category, there are further subcategories that include distraction-based methods, compression-based methods, growth guidance, and hybrid. Most of the alternatives to spinal fusion that are used today for the management of pediatric scoliosis fall under the distraction-based method category, which includes traditional growing rods, magnetically controlled growing rods (MCGRs), and VEPTRs. Combination surgical methods utilize limited spinal fusion in addition to growth-friendly methods. Indications for each surgical method depend on scoliosis etiology, Cobb angle, remaining spinal growth, and risk of curve progression, among other factors. 

MCGRs are one of the newer surgical interventions for the management of scoliosis, mainly EOS, along with the even newer vertebral body tethering (VBT) method. The indications and surgical procedure for MCGRs are similar to those of growing rods, but they do not require repetitive surgeries for the lengthening or shortening of the rods. Instead, rod lengths can be adjusted transcutaneously with an external remote control, which can be done in an outpatient setting. A systematic review conducted on the outcomes and complications associated with MCGRs found that at 2.5 years' follow-up, the curve degree improved from 65 to 35 degrees and the total complication rate was 45% [48]. The most commonly identified complications were unplanned revision surgery, implant failure, and rod breakage/foundation failure. There have since been several studies reporting implant failure and rod breakage, which led to design changes, but the full issue has yet to be completely addressed [48]. Given these known complications and structural design issues, traditional growing rods may be a better initial option for now. By contrast, VBT is a compression-based surgical method that allows for curve correction without fusion by using the patient’s own spinal growth to improve the degree of curvature after surgery [49]. During the procedure, screws are inserted laterally into each vertebral body and a cable is used to connect the screws. Immediate curve correction occurs via compression of the cable between the screws, and additional correction occurs via vertebral body growth modulation according to the Hueter-Volkmann principle. A systematic review of VBT outcomes found that the mean preoperative main thoracic curve was 49 degrees, which corrected to 24 degrees in the first postoperative imaging, and that lumbar curves underwent spontaneous correction from 25 degrees to 7 degrees in two years [49]. The complication rate was found to be 18%, with pulmonary (mainly pneumothorax and pleural effusion) and instrumentation-related being the most common. Reoperations were required in 10% of cases [49]. Published data on outcomes and complications remain sparse, and findings were variable between studies. Furthermore, a 2020 study comparing VBT to definitive fusion found that correction can be better maintained with fusion with less revision procedures and similar health-related quality of life (HRQOL) outcomes [50]. 

In children with AIS, posterior fusion is thought to be the most frequently used surgical approach (75%), and despite new technologies, surgical techniques, and modern instrumentation, complication rates, as indicated by surgeon reports, have remained relatively constant [45,46]. Generally, between 5% and 23% of all AIS patients experience surgical complications. A 2015 study by Martin et al. that analyzed a multicenter pediatric surgery registry found that the 30-day unplanned readmission rate after pediatric spinal deformity surgery was 3.96% and that the top reasons for readmission were wound complications and gastrointestinal disturbances [51]. In addition, they determined that isolated anterior spinal fusions, structural pulmonary abnormalities, and an American Society of Anesthesiologists class of 3 or 4 were independently associated with readmission after surgery [51]. Another similar study found that the 90-day readmission rate was 8% for all types of scoliosis, with the most common causes being wound complications and pulmonary conditions [52]. In addition, based on the National Inpatient Sample (NIS) database and metrics found in other literature, it has been reported that the complication rate due to pulmonary dysfunction post spinal fusion is up to 3.5% [53,54]. Interestingly, there are conflicting reports as to whether these postoperative pulmonary issues have a direct correlation with preoperative PFTs, implying that further analysis is required in the investigation of the association between PFTs and post-operative surgical outcomes [45]. 

Currently, the primary goal in the treatment of EOS and AIS is to maximize the growth of the spine and thorax by controlling spinal deformity, with the aim of promoting normal lung development and pulmonary function [41]. This refurbished goal in IS treatment allows for surgeons to address the overall health of children and subsequently lead to the greatest improvement in their QOL. This also allows for the re-evaluation of non-surgical techniques, such as bracing and casting, to play a greater role in scoliosis treatment. If the scoliosis is not severe enough in its progression, the non-operative treatment is indicated as the corrective procedure for both EOS and AIS [55,56]. This allows for improvement in the curve progression of the spine, while also maintaining the potential of the thoracic cage to expand normally and develop adequate lung volumes. This does not mean, however, that surgical intervention should not be relied upon or can be completely replaced by non-operative measures. In most cases where the scoliosis is moderate to severe (cases in which the Cobb angle is too large to be corrected by a brace or cast), surgical management should be considered [26]. 

There are many factors that affect pulmonary function postoperatively in children with EOS and AIS. One major component is the type of surgical intervention. Although most surgical interventions are relatively safe and aim to correct the spinal deformity and its sequelae, studies have demonstrated mixed effects of surgical approach on pulmonary function, even in the context of a reduced primary curvature, as increases, decreases, and no changes in pulmonary function have all been seen [26,44,57,58]. There also seems to be a significant impact on postoperative outcomes depending on how early surgical intervention was implemented in the patient’s care. Some literature highlights that early surgical intervention may result in a short trunk and stunted growth of the thorax and lungs, while others have highlighted that early intervention through VEPTR or mechanically controlled growing rods can lead to significant increases in lung volume in most patients, as evidenced by increases in the FVC over time [20,29,43,59-61]. One must keep in mind, however, that the “golden” period for both thoracic spine and cage growth occurs between birth and eight years of age. This golden period also coincides with lung development. Another major component is the variable pathophysiology of lung disease in these growing children, which is not yet fully understood. Therefore, it is extremely important for orthopedists to preserve both thoracic growth and lung volume with respect to any surgical intervention during this critical period of life [62].

Lastly, in addition to correctional treatment, to maximize patient reported outcomes, care teams should also administer and follow up with quantifiable methods of assessing the QOL of both the children and their parents/guardians/caretakers postoperatively. Even in cases where pulmonary function and daily living significantly improved after surgical intervention, other important aspects regarding QOL can be negatively impacted as a result. Chang’s group highlighted that even though they observed significant improvement in these two QOL domains, there was also a significant decline in the domains of pain, emotion, and satisfaction [39]. Similarly, a multicenter study published by Matsumoto et al. [63] reported significant improvement in pulmonary function and financial subdomain postoperatively in a scoliosis-specific patient-reported outcome measure (PROM). However, satisfaction, pain, transfer, and parental satisfaction trended toward decrease in scores at two-year assessments [63]. 

**Figure 1:**
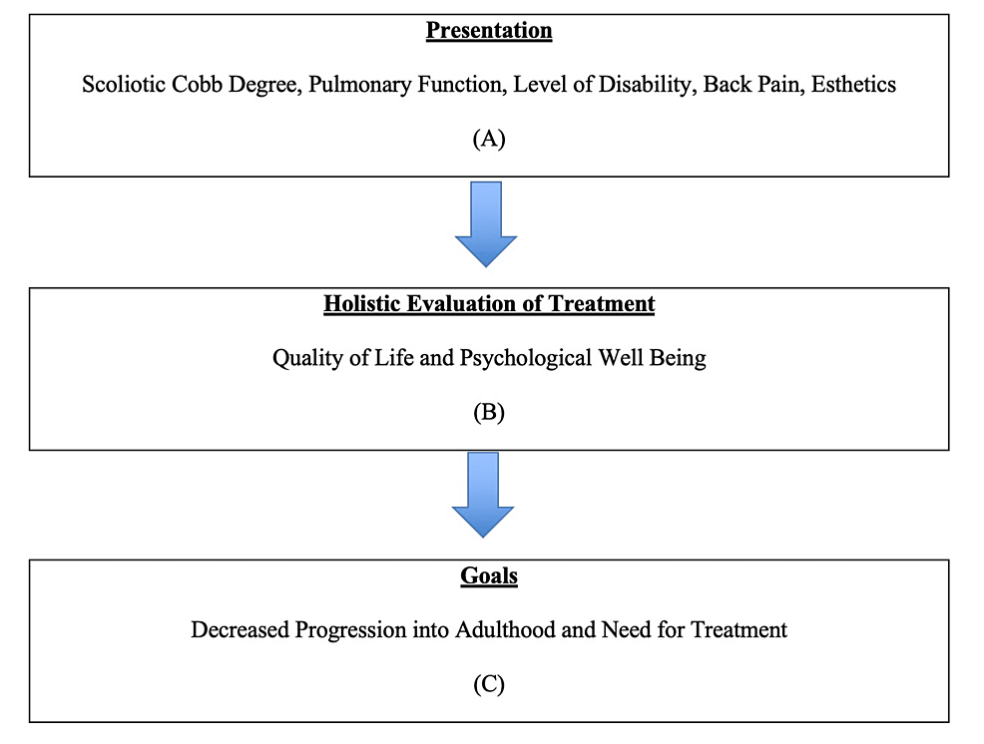
Holistic treatment schema of EOS and AIS. The current early onset scoliosis (EOS) and adolescent idiopathic scoliosis (AIS) treatment includes all domains listed in the figure. Proposed here is a holistic management method that involves (A) identifying and correcting symptoms of the primary pathology, (B) domains that can be longitudinally assessed to evaluate a holistic treatment efficacy, and (C) goals of the said treatment. The arrows indicate a stepwise progression. Image Credits: Emmanuel Mbamalu.

This evidence supports the increasing need for medical care teams to utilize PROMs in the ongoing treatment of children with EOS and AIS for not just lung dysfunction but overall health, with lung dysfunction being a major component. The 24-Item Early Onset Scoliosis Questionnaire (EOSQ-24) and Early Onset Scoliosis Self-Report Questionnaire (EOSQ-SELF) are valid methods of collecting this data through both a proxy and the children themselves (respectively) that care teams can easily integrate into their plans by administering during a visit [64]. There also exist a variety of HRQOL questionnaires/surveys that may assist in providing more well-rounded care in the treatment of children with AIS that are available online [65]. These practices allow the treatment of patients with EOS and AIS to incorporate lung dysfunction in a holistic, patient-centered view that improves overall patient outcomes, as shown in Figure 1. 

## Conclusions

This review aimed to emphasize the necessity of addressing pulmonary dysfunction as a major concern in the treatment of children with EOS and AIS. Current literature has highlighted the complexity of the pathophysiology of lung disease in EOS and AIS. In addition to reduced lung volume, weakness in respiratory muscles and the central nervous system can lead to decreased pulmonary function for patients with EOS and AIS. These deficits are made apparent by decreased FVC and FEV1 values on PFTs. Non-operative and surgical interventions offer possible corrections for the curve progression while maintaining potential for maximizing lung volume by allowing for growth of the thoracic cage. As a part of treatment, long-term management of children with IS should include the assessment of QOL domains. This can be achieved through specific PROMs in order to ensure well-rounded and holistic care. These general principles are now becoming central concepts in the modern treatment and care plan of children with EOS and AIS.

It is proposed that future studies further examine the role of pulmonary dysfunction in EOS and AIS. As our understanding of their pathogenesis increases, we will better be able to improve care and outcomes for children afflicted by these diseases. The role of PFTs should also be established in the management of IS. Although there have been conflicting reports on the usefulness of PFTs in the long-term care of patients, by utilizing them early and in conjunction with PROMs, it is possible that they can serve as a guideline to improve the QOL post intervention. Finally, awareness of lung diseases in children with EOS and AIS should be increased. Raising awareness may address the primary purpose of this paper by leading to more investigation regarding the topic.
